# Expanded roles of community health workers to sustain malaria services in the Asia-Pacific: A landscaping survey

**DOI:** 10.1371/journal.pgph.0003597

**Published:** 2024-08-14

**Authors:** Monnaphat Jongdeepaisal, Massaya Sirimatayanant, Panarasri Khonputsa, Phone Si Hein, Laura Buback, Naomi Beyeler, Amita Chebbi, Richard J. Maude

**Affiliations:** 1 Mahidol Oxford Tropical Medicine Research Unit, Faculty of Tropical Medicine, Mahidol University, Bangkok, Thailand; 2 Centre for Tropical Medicine and Global Health, Nuffield Department of Medicine, University of Oxford, Oxford, United Kingdom; 3 Asia Pacific Malaria Elimination Network (APMEN), Singapore, Singapore; 4 Global Health Group, UCSF Institute for Global Health Sciences, San Francisco, California, United States of America; 5 Harvard TH Chan School of Public Health, Harvard University, Boston, Massachusetts, United States of America; 6 The Open University, Milton Keynes, United Kingdom; Makerere University School of Public Health, UGANDA

## Abstract

Malaria Community Health Workers (CHWs) in the Greater Mekong Subregion (GMS) are an important component of malaria elimination efforts. As malaria declines with intensified efforts to eliminate by 2030, expanding their roles beyond malaria could help to sustain funding and provision of malaria services at the community level. Evidence of how programmes have implemented and managed CHWs performing both malaria and non-malaria roles across the Asia-Pacific region can provide insight into the viability of this strategy. A short survey was distributed to national malaria programmes and implementing organizations in the Asia-Pacific region in 2021–2022. The survey identified CHW programmes in the region, and collected information on malaria and non-malarial services provided by CHWs, characteristics of each identified programme, and the impact of COVID-19 on these programmes. 35 survey responses identified 28 programmes in 14 countries. The most frequently reported services provided by malaria CHWs were health promotion and education for malaria (13/14 countries) and other diseases (11/14); and COVID-19 related activities (10/14). Most programmes were financed wholly through donor funding (18/28 programmes), or donor plus government funding (6/28). Of 21 programmes which performed programme evaluation, only 2 evaluated their impacts on diseases beyond malaria. Declining donor funding, and COVID-19 related travel and activity restrictions were identified as implementation challenges. CHWs across the Asia Pacific provide a range of health services with malaria and are resilient under changing public health landscapes such as the COVID-19 pandemic. Further investigation into the impact of additional roles on malaria CHW performance and targeted health outcomes is needed to verify the benefits and feasibility of role expansion. As the GMS approaches elimination, and funding declines, verifying the cost effectiveness of malaria CHW programmes will be vital to persuade donors and countries to invest in malaria CHWs to sustain malaria services, and strengthen community-based health care.

## Introduction

In the Greater Mekong Subregion (GMS), malaria incidence and mortality have reduced substantially in the past 20 years. Progress has been made through tailoring malaria interventions to reach populations-at-risk in rural areas [1]. In such remote settings, community health workers (CHWs) have been leveraged to improve access to care for malaria services. While there is variability in defining these worker groups, CHWs are generally individuals selected by, or working within, their communities, often receiving training to implement specific health interventions and perform roles in healthcare delivery, as outlined by the World Health Organization (WHO)(13). In the GMS, malaria-specific CHWs are often referred to as Village Malaria Workers (VMWs) and play a significant role by providing prompt malaria testing among other malaria-related services [2–4]. With the increasing emergence of multidrug resistant malaria, the disease remains a serious threat in the region [5]. This has led to countries prioritizing malaria elimination to ensure that the region achieves its elimination target by 2030 [6].

CHWs have been recognized as an essential workforce in primary healthcare systems across the world. These frontline workers provide health services in their communities and support public health facilities in increasing accessibility of health services in remote areas [7]. Their roles are often determined by context specific health priorities or targets set by governments or donors [7]; in malaria endemic areas, National Malaria Control Programmes (NMCPs) may recruit and train community members to provide malaria services. NMCPs may also draw on existing CHW schemes to provide malaria services in addition to basic health services. As malaria declines in the GMS, provision of malaria services by these workers may be less prioritized and supported despite wide acceptance of their benefits [8]. To maintain momentum towards elimination, leveraging malaria CHWs in vertical malaria programmes to perform additional health services has been proposed as a strategy by GMS countries to sustain funding and provision of malaria services at the community level [1, 9]. In public health, “vertical programme” refers to health programmes that selectively target interventions for specific health issues and are not fully integrated into the health system, while “horizontal programmes” deliver health services through publicly financed health systems and are commonly referred to as comprehensive primary care. Here, the term vertical is used to elaborate the roles of malaria CHWs, currently under a malaria-specific (vertical) programme, which could be expanded beyond malaria. GMS countries, including Cambodia, Lao PDR, Myanmar, Thailand and Vietnam, could learn from the wider regional context about existing CHW programmes and the package of services they provide alongside malaria to inform potential expansion strategies. Evidence of how active programmes in the Asia Pacific implement and manage CHWs performing both malaria and non-malaria roles is thus needed.

This article reports findings from an online survey aiming to identify and characterize expanded malaria CHW programmes in the Asia Pacific region. The survey collected information on malaria and non-malaria services provided by CHWs, characteristics of each identified programmes, and the impact of the COVID-19 pandemic on the programmes in varied implementation contexts.

### Study context

The study is part of operational research supported by the Global Fund to Fight AIDS, Tuberculosis and Malaria, titled *Sustaining village health worker programmes with expanded roles in the GMS (*2021–2023). The landscape analysis component of this operational research comprises of a systematic review (protocol registered on PROSPERO CRD42021250639) [10], this survey, and follow-up interviews with implementers. The aim of the landscape analysis is to comprehensively document the current status and challenges of existing malaria CHW programmes with expanded roles in the region. Specifically, this online survey (2021) was conducted in parallel with a systematic review (2021–2023) in order to fill gaps identified in the review [10] and identify implementers for further interviews.

The study is a collaboration between Mahidol-Oxford Tropical Medicine Research Unit (MORU) and the Asia Pacific Malaria Elimination Network (APMEN), a regional network of countries and stakeholders committed to eliminating malaria in the Asia Pacific by 2030, and the Malaria Elimination Initiative, University of California, San Francisco, a research institution working with malaria endemic countries to advance malaria policy and practice. Results from each component of the landscaping analysis will be published separately.

## Methods

### Survey development

A questionnaire was developed to collect updated information on programmes with CHWs performing roles beyond malaria services, hereinafter referred to as “malaria CHWs”, in the Asia Pacific. For this survey, CHWs are defined as a group of healthcare workers selected by, or working in, their communities, and may receive training to perform specific health interventions and/or roles related to healthcare delivery (WHO) [11]. Use of a wider definition of malaria CHWs is intended to capture different groups of community-based health workers beyond VMWs that may exist across, or within, the same country in the Asia-Pacific region. Therefore, it is important to note that the malaria CHWs discussed in this study cover a wide array of health workers, some of whom may not fit the typical classification of CHWs. Additionally, this study uses the term programme to refer to health programmes that implement any health activities and manage one or more CHW cadres providing malaria services and beyond, hereinafter referred to as malaria CHW programme or programme.

Survey questions were informed by the initial results of a systematic review [10], and were revised based on pilot-testing with a focus group of potential respondents. The full list of survey questions can be found in **S1 Appendix**. The survey was comprised of 30 questions under three topics: (1) general information about the respondent and programme, (2) type of CHWs, the malaria and non-malaria services they provide, and (3) implementation characteristics of the programmes such as training, supervision, financing, collaboration, and evaluation activities. Open-ended questions about how the COVID-19 pandemic had affected each programme were also included. Respondents were asked to respond based on how the programme was operating at the time of the survey, or, if no longer active, how it operated prior to discontinuation. Respondents were limited to submitting responses for up to 3 programmes per 1 survey submission, a constraint of the Jisc software used for the online survey. At the end of the survey, respondents were asked to identify a programme representative to be interviewed further.

### Survey distribution

The online survey was voluntary and distributed to managers and staff of NMCPs in 23 Asia-Pacific countries (22 APMEN partner countries, and Iran), and other organisations implementing or supporting malaria CHW programmes. Programmes were identified from among APMEN partners and observers, survey responses, and supplementary searches. Target respondents were staff involved in managing or implementing malaria CHW programmes in Asia-Pacific countries. Target respondents were invited to participate by email with a link to the online survey, and in which they were informed that they may forward the invitation to an eligible respondent within their organisation. The survey was launched on 15^th^ October 2021, with follow-up email reminders being circulated throughout the data collection period until the closing date on 31^st^ January 2022. The survey was self-administered in English using the Jisc online surveys platform.

### Data processing and analysis

Survey responses were processed and analysed in Microsoft Excel. Codes were created to identify each individual programme. To simplify the analysis, responses identifying the same programme and/or same CHW cadres in the same programme were combined. Any discordant responses about a programme or CHW cadre by multiple respondents were re-verified with the respective programmes. Respondents could select more than one response for questions that allowed multiple answers, therefore, survey findings present frequency counts of selected responses by programme. These frequencies were used to summarize categorical data. The proportion of CHW cadre(s) performing an identified role within each country was calculated as a percentage. The proportion of programmes performing monitoring and evaluation activities within each country was also calculated in percentages. Free text responses regarding programme challenges and COVID-19 impacts were coded thematically using NVivo software. GraphPad Prism was used to visualize the findings.

### Ethics statement

This study was approved by the Oxford Tropical Research Ethics Committee (OxTREC reference no. 534–21). All respondents provided formal written informed consent prior to taking the survey by ticking a box in the online form to indicate that they had understood the information about the study, confirmed that they met the inclusion criteria, and agreed to take part. Survey responses did not routinely record names, email addresses, or IP addresses of respondents, but collected contact information of programme representatives willing to be interviewed further. To maintain confidentiality of the identified representative, their contact information was not shared beyond the study team (MS, MJ, and LB), nor used in data analysis and reporting.

## Results

**Fig 1:**
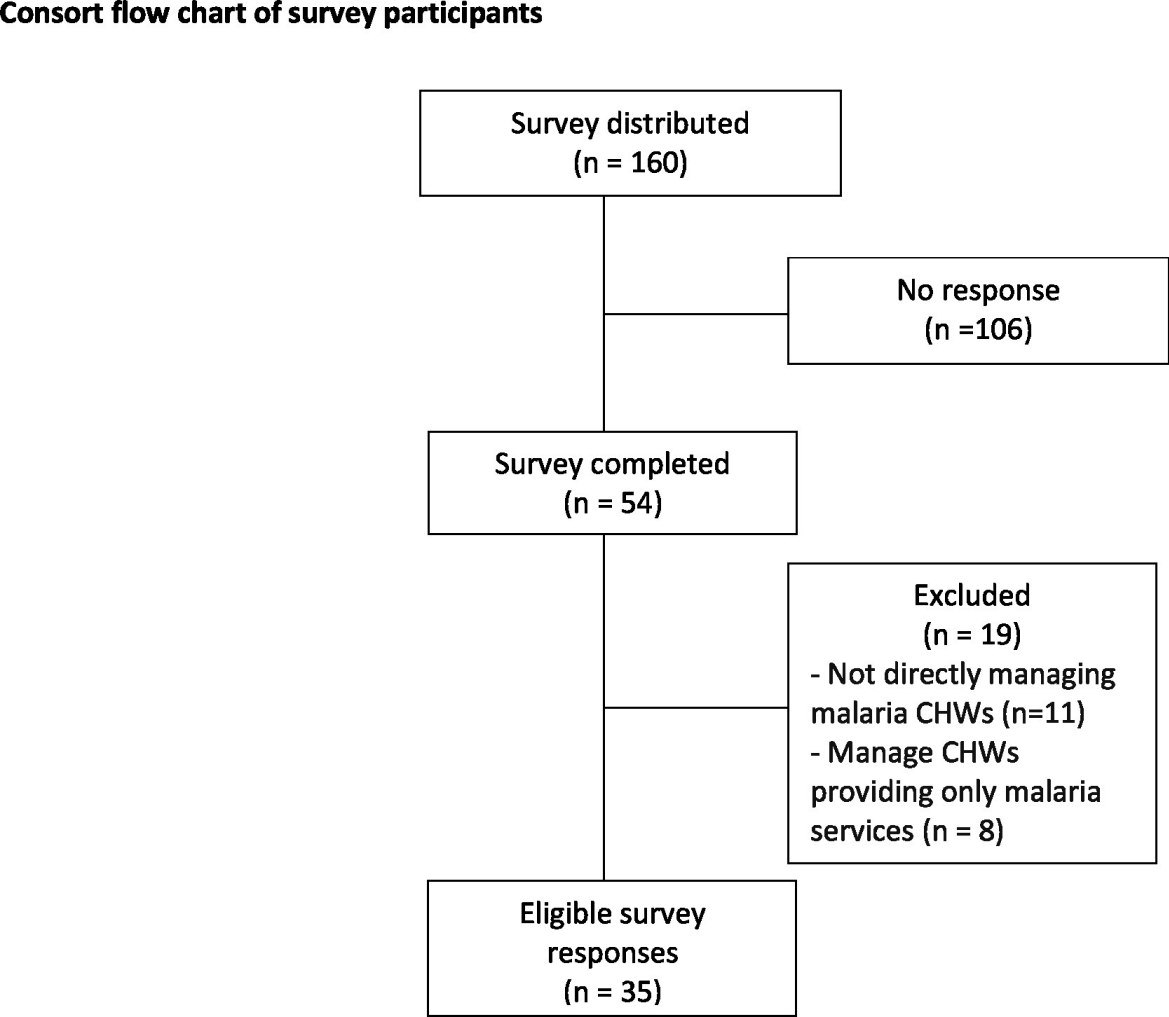
Consort flow chart of survey participants.

The survey received 54 responses from 28 programmes in 16 countries, with 10 respondents identifying as international organisations based in multiple countries. **Fig 1** illustrates the consort flow chart of survey participants, and **Table 1** provides baseline characteristics of survey respondents that identified included programmes. Baseline characteristics of excluded survey respondents can also be found in **S2 Appendix**.

**Table 1:** Baseline characteristics of eligible survey respondents.

	Respondents		Programmes	
	(n = 35)		(n = 28)	
	n	%	n	%
Afghanistan (AFG)	1	2.9	1	3.6
Bangladesh (BNG)	1	2.9	1	3.6
Bhutan (BTN)	1	2.9	1	3.6
Cambodia (KHM)	5	14.3	4	14.3
India (IND)	2	5.7	2	7.1
Lao PDR (LAO)	1	2.9	1	3.6
Malaysia (MYS)	2	5.7	1	3.6
Myanmar (MYM)	9	25.7	8	28.6
Nepal (NPL)	2	5.7	2	7.1
Papua New Guinea (PNG)	1	2.9	1	3.6
Solomon Islands (SLB)	1	2.9	1	3.6
Sri Lanka (LKA)	3	8.6	1	3.6
Thailand (THA)	4	11.4	2	7.1
Viet Nam (VNM)	2	5.7	2	7.1
Organisation type				
Academic/Research Institution	4	7.4	2	7.1
International (non-governmental) organization	16	29.6	13	46.4
Non-governmental organization	12	22.2	3	10.7
Government/National Malaria Control Programme	16	29.6	8	28.6
Private sector	1	1.9	1	3.6
Other^2^	4	7.4	1	3.6
Work position				
Director/manager	25	46.3		
Project/programme officer	13	24.1		
Technical advisor/Specialist	16	29.6		

Note:

^1^ Respondents may work in organisations that operate in many countries such as international organisation (IO) or international non-governmental organisations (INGOs).

^2^ Others represent respondents identifying themselves as community-based organisations or former staff of any organisation types.

Of the 54 responses, 19 were screened out due to ineligibility for 1) respondents not directly managing malaria CHWs (n = 11) or 2) programmes managing CHWs that only provided malaria services (no additional services; n = 8). Among 35 eligible responses, 28 programmes were identified in 14 countries. Five programmes received multiple responses which were combined. Survey respondents were programme managers/directors (n = 17), programme staff (n = 9), and technical advisors/specialists (n = 9) including academics, entomologists, epidemiologists, and clinicians. Survey findings did not cover all Asia-Pacific countries with no responses from 7 out of 21 APMEN country partners. Description of ‘other’ responses selected in the survey reported in the following tables and figures can also be found in **S2 Appendix**.

### Identification and characterization of programmes with expanded roles beyond malaria

Twenty-four programmes are still active: 11 operating at national level, 12 sub-national, and one ad-hoc programme established specifically for COVID-19 emergency response. The majority of programmes identified were in Myanmar (8) and Cambodia (4), reflecting the implementation structures in both countries whereby malaria CHW programmes are implemented by partners such as civil society organisations (CSOs) and non-governmental organisations (NGOs). Four programmes were no longer active: a short pilot (KHM2), a programme that transitioned into another programme (KHM3) and 2 discontinued programmes (MMR3 and PNG). A full list of identified programmes and their characteristics, and CHW cadres is in **Table 2**.

**Table 2:** Programmes with CHWs providing health services beyond malaria in the Asia Pacific.

Country name (abbrev.)	Implementer/Programme name	CHW cadre(s)	Programme scale (number of health facilities/posts and CHWs)	Programme duration, status
**Afghanistan (AFG)**	Community Health Worker Programme	Community Health Workers (CHW)	Almost 2000 Health posts/mobile health units, BHCs, CHCs, district and provincial hospitals >50,0000 CHWs (>30,000 MoPH and about 20,000 International Red Cross Society)	More than 5 years, active
**Bangladesh (BNG)**	Bangladesh Rural Advancement Committee (BRAC) in partnership with National Malaria Control Programme	(1) Shasthya Sebikas (SS), Community Health Volunteers (2) Shasthya Kormi (SK), Health Workers	71 Health facilities/posts 4252 volunteers/workers	More than 5 years, active
**Bhutan (BTN)**	Vector-Borne Disease Control Programme	(1) Health Assistant (HA) (2) Village Health Worker, members of Community Action Group (CAG)	650 HAs deployed at all Primary Health Centres (184) 1,053 VHWs deployed in every village [12]	More than 5 years, active
**India (IND1)**	Malaria Elimination Demonstration Project (MEDP) in Mandla District, Madhya Pradesh	(1) Village Malaria Worker (VMW) (2) Malaria Field Coordinators (MFC)	298 health facilities/posts 260 workers (25 Malaria Field Coordinators and 235 Village Malaria Workers) [13]	1 to 5 years, active
**India (IND2)**	National Health Mission of Chhattisgarh	Mitanin (MTN)	1 medical college, 1 district hospital, 10 community health centre, 45 Primary health centre, 350 sub health centres >4000 Mitanin	More than 5 years, active
**Cambodia (KHM1)**	Catholic Relief Services (CRS)’s RAI3E, TB Programme and Resilient Sustainable System for Health Programme	(1) Village malaria worker (VMW) (2) Mobile malaria worker (MMW) (3) Village Health Support Group (VHSGs) (4) Community DOTs Watchers (CW)	80 health facilities/posts 774 workers (breakdown unspecified)	More than 5 years, active
**Cambodia (KHM2)**	Health Poverty Action (HPA)’s COVID-19 Emergency Response	Mobile Malaria Worker (MMW)	Approx. 20 health facilities/ posts N/A workers due to inactivity of programme	6 months to less than 1 year, no longer active
**Cambodia (KHM3)**	Population Services International (PSI)’s Worksites Programme	Mobile Malaria Worker (MMW)	<20 Operational Districts 274 MMWs	More than 5 years, no longer active
**Cambodia (KHM4)**	National Center for Parasitology, Entomology and Malaria Control (CNM)’s Village Malaria Worker Project	(1) Village malaria worker (VMW) (2) Mobile malaria worker (MMW) (3) Village Health Groups (VHG)	>1000 health facilities/posts Number of workers not specified	More than 5 years, active
**Lao PDR (LAO)**	National Malaria Control Programme’s Center for Malariology, Parasitology, and Entomology (CMPE)	(1) Village Malaria Worker (VMW) (2) Forest Malaria Worker (FMW)	260 health facilities/posts 2199 workers (breakdown unspecified)	More than 5 years, active
**Sri Lanka (LKA)**	Anti-Malaria Campaign (AMC)	(1) Public Health Field Officers (PHFO) (2) Health Entomology Officers (HEO) (3) Spray Machine Operators (SMO) (4) Public Health Laboratory Technicians (PHLT) (5) Public Health Inspectors (PHI)	AMC-HQ and 27 RMO offices Approx. 80 workers (PHFO, HEO, and SMO)	More than 5 years, active
**Myanmar (MMR1)**	Shoklo Malaria Research Unit (SMRU)’s Malaria Elimination Task Force (METF)	(1) Malaria Post Worker (MP) (2) Community Health Worker (CHW)	Approx. 1000 health facilities/posts >900 workers (breakdown not specified)	More than 5 years, active
**Myanmar (MMR2)**	Malaria Consortium (MC)’s expanding rural communities’ access to health services in Myanmar programme	(1) Malaria Volunteer (MV) (2) Integrated Community Malaria Volunteers (ICMV)	Unspecified number of health facilities/posts 120 workers (breakdown unspecified)	1 to 5 years, active
**Myanmar (MMR3)**	Population Services International (PSI)’s Community Health Services Programme	Community Health Services Provider (CHSP)	Unspecified number of health facilities/posts N/A workers due to inactivity of programme	1 to 5 years, no longer active
**Myanmar (MMR4)**	Save the Children’s Regional Artemisinin Initiative Programme in Myanmar	Integrated Community Malaria Volunteer (ICMV)	Health facilities/post not involved in programme 235 ICMVs	1 to 5 years (for ICMVs under regional grants) More than 5 years (for ICMVs under country grants), active
**Myanmar (MMR5)**	Malteser International (MI)’s Regional Artemisinin Initiative Programme in Myanmar	Integrated Community Malaria Volunteer (ICMV)	4 Health facilities/posts 182 ICMVs	1 to 5 years, active
**Myanmar (MMR6)**	Medical Action Myanmar (MAM)’s Community Health Worker Programme	(1) Community Health Workers (CHW) (2) Integrated Community Malaria Volunteer (ICMV) (3) Auxiliary midwives (AM)	Workers operate from their homes in 2000 villages (not associated to health facilities/posts) Approx. 2000 workers (unspecified breakdown)	More than 5 years, active
**Myanmar (MMR7)**	Myanmar Council of Churches (MCC)’s Community Based Malaria Prevention and Control Project	(1) Village Health Volunteer (VHV) (2) Integrated Community Malaria Volunteer (ICMV)	532 health facilities/post 532 workers (unspecified breakdown)	More than 5 years, active
**Myanmar (MMR8)**	ALIGHT Regional Artemisinin Initiative Programme	(1) Integrated Community Malaria Volunteer (ICMV) (2) Microscopist (MCS) (3) Community Mobilizer (CM)	45 health facilities/posts 729 workers (unspecified breakdown)	More than 5 years, active
**Malaysia (MYS1)**	Malaria Ambassador and Primary Health Care Volunteer Programme	(1) Malaria Ambassador (MA) (2) Primary Health Care Volunteer (PHCV)	368 health facilities/posts 482 workers (unspecified breakdown)	More than 5 years, active
**Nepal (NPL1)**	Community Based Testing in National Malaria Elimination Programme	Malaria Village worker (MVW)	55 health wards 55 MVWs	6 months to less than 1 year, active
**Nepal (NPL2)**	Health Assistants in the National Malaria Elimination Program	Health Assistant (HA)	55 health wards 210 HAs	1 to 5 years, active
**Papua New Guinea (PNG)**	Village Malaria Assistants Network from the Lihir Malaria Elimination Programme	Village Malaria Assistant (VMA)	8 health facilities/posts 84 VMAs (2020, pre-discontinuation)	1 to 5 years, no longer active
**Solomon Islands (SLB)**	Vector Borne Disease Control Programme	(1) Community Malaria Microscopists (CMM) (2) Civil Society Organizations (CSO) (cadre not specified)	> 200 health facilities/posts > 1000 for LLIN distribution (breakdown not specified)	6 months to less than 1 year, active
**Thailand (THA1)**	Mae Tao Clinic	(1) Community Health Workers (CHW) (2) Medic (MD)	1 health facility/post 15 CHWs 53 MDs	More than 5 years, active
**Thailand (THA2)**	Division of Vector Borne Disease (DVBD)’s Malaria Elimination Programme	(1) Malaria Post Worker (MPW) (2) Malaria Volunteer (MV)	450 health facilities/posts 450 workers (breakdown not specified)	More than 5 years, active
**Vietnam (VNM1)**	Health Poverty Action (HPA)’s Regional Artemisinin-resistance Initiative Programme	(1) Village Health Worker (VHW) (2) Malaria Volunteer (MV) (3) Malaria Coordinator (MC)	39 health facilities/posts 403 workers (breakdown unspecified)	1 to 5 years, active
**Vietnam (VNM2)**	National Institute of Malariology, Parasitology and Entomology (NIMPE)’s National Malaria Control and Elimination Programme	Village Health Worker (VHW)	All health stations in 63 provinces Unspecified number of VHWs	More than 5 years, active

### Health services provided by malaria CHWs

**Fig 2:**
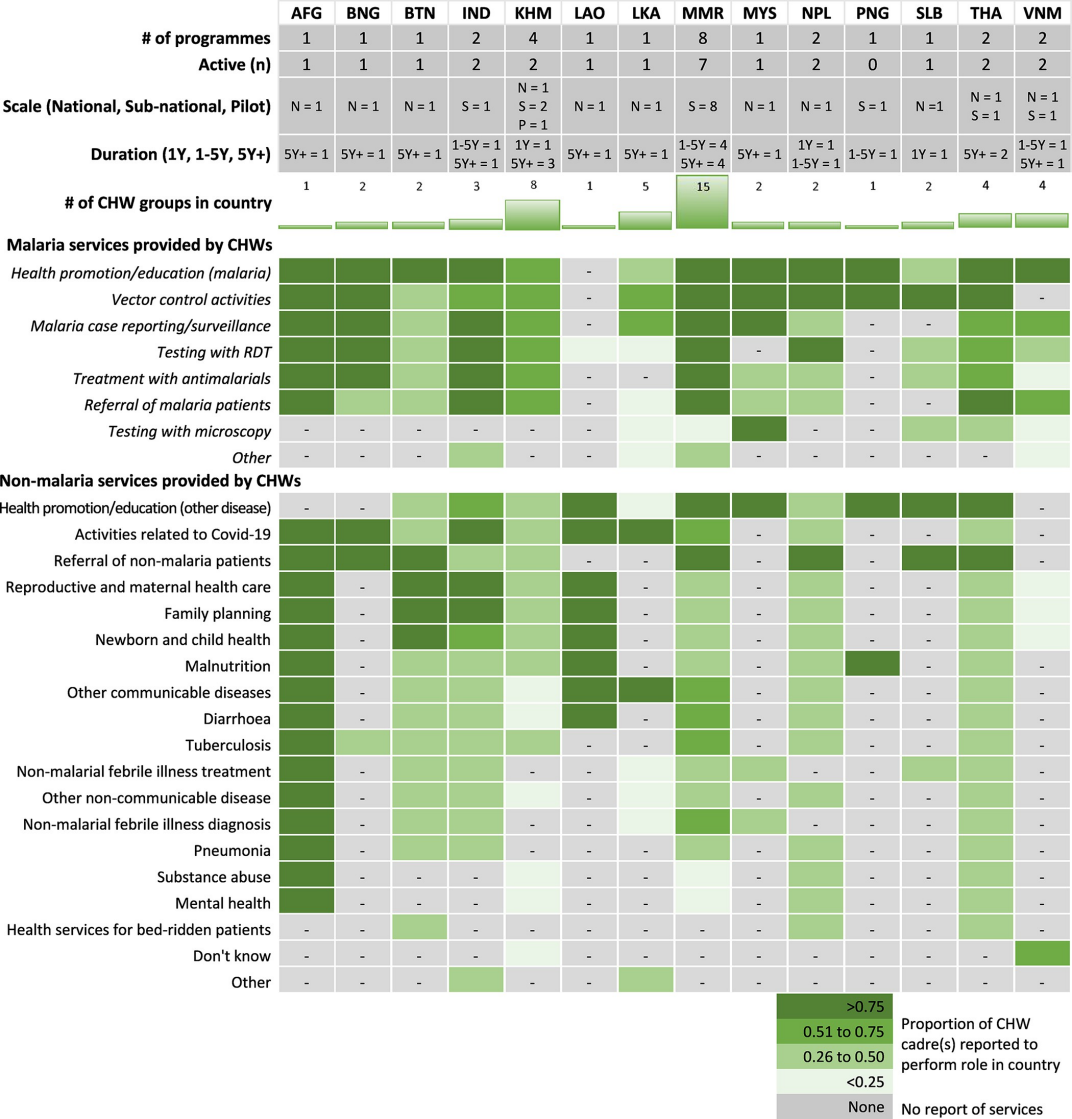
What health services do malaria CHWs provide in each country?

52 malaria CHW cadres were identified from the 28 eligible programmes. Most programmes managed 1 (n = 11) or 2 (n = 11) types of CHW cadres with only 6 programmes managing between 3 and 5 CHW cadres. **Fig 2** summarises reported malaria and non-malaria services provided by CHW cadres at the country level. Each cadre is referred to throughout this paper using an abbreviation shown in the first column of **Fig 2**. In this, the first 3 letters indicates the country followed by a number in roman numerals for the specific programme, for example LAO-I is the Village Health Volunteer programme in Lao PDR. Health promotion or education for malaria was the most reported malaria-related service provided by CHW cadres (44 of 52 cadres), in 13 of 14 countries, followed by vector control, malaria case reporting and/or surveillance activities (both performed by 39 CHW cadres), and malaria testing with rapid diagnostic tests (38 cadres). Testing for malaria by microscopy was the least frequently reported malaria-related service, performed by 9 cadres of malaria-specific workers such as malaria coordinators (SLB1), malaria ambassadors (MYS1), integrated community malaria volunteers (ICMVs, MMR2); microscopists/lab technicians (LKA1, MMR8, SLB), medics (THA1) and other general health workers (THA1, MYS1).

Health promotion and education about other diseases was the most identified non-malaria service performed by 31 CHW cadres in 11 of 14 countries. This was followed by activities relating to COVID-19 (27 CHW cadres in 10 countries), and referral of non-malaria patients (31 cadres in 9 countries). Countries with CHWs that were not part of a vertical malaria programme reported covering the largest number of non-malaria roles: CHWs and medics in THA1 (17 out of 17 selectable roles), CHWs in AFG (15 roles), Health Assistants (HA) in BTN and NPL (15), and CHWs and auxiliary midwives in MMR6 (14). However, the response from MMR6 also indicated that the ICMVs, malaria-specific volunteers with expanded roles, also performed 14 non-malaria roles equal to the CHWs and auxiliary midwives within the same programme.

### Programme characteristics

#### Programme training and supervision

**Fig 3:**
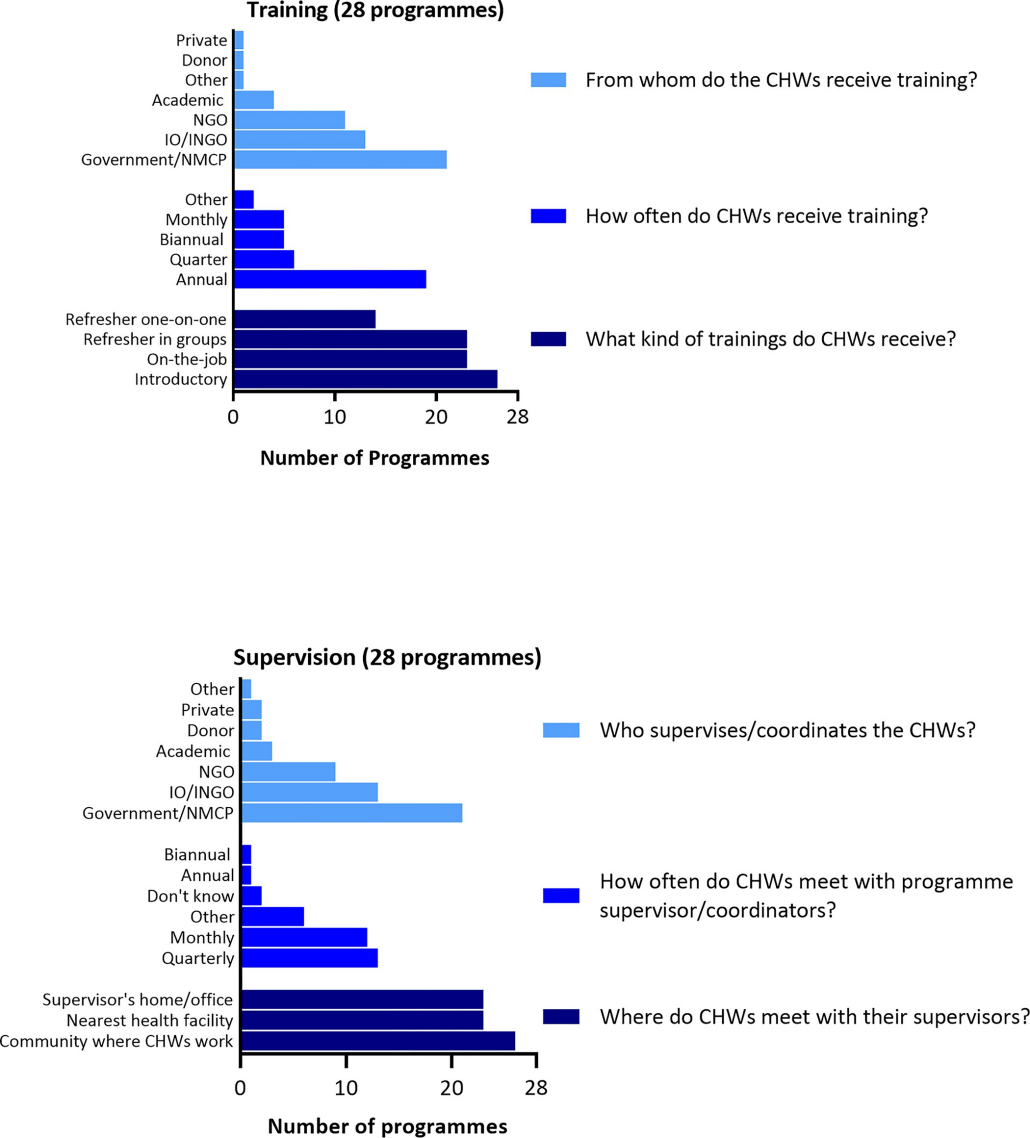
Programme training and supervision.

Programme implementation characteristics were collected and reported at the programme level. All programmes (n = 28) reported providing training and supervision; specific characteristics of each activity are presented in **Fig 3**. Twenty-six programmes reported providing introductory training, 23 on-the-job training and refresher training in groups for malaria CHWs, and 14 provided one-on-one style refresher training. Eight programmes specified that some of the training sessions are provided online. Most programmes (19) provided training annually, 2 programmes indicated that training may be hosted on an ad hoc basis, either based on volunteer needs (MMR5), or upon recommendation or availability of funds (AFG). Supervisory sessions were provided more frequently than training, with 13 programmes providing supervision quarterly and 12 monthly. Three programmes provided supervision more than once a month (IND1, LKA1, and THA1), and 3 on an ad hoc basis depending on accessibility to the area (MMR1 and MMR5) or supervisory needs (NPL1). In 26 programmes, supervisory visits happened within the community where malaria CHWs work, but may also be hosted at the health facility (21) and/or at the supervisor’s home (9). Governmental organisations or NMCPs were reported to be the principal provider for both training and supervision in 21 programmes, followed by international organizations/non-governmental organizations (IOs/INGOs) (13). Local NGOs provided training for 11 programmes, and supervision for 9.

Challenges to providing training and supervision were identified by 22 programmes. Many programmes cited lack of funding as the main limitation to providing training (15), with less than half citing other challenges such as lack of available trainers (8), updated training materials and/or guidelines (5), and the COVID-19 pandemic limiting in-person activities (5). As supervision was often provided within the communities, lack of access to, or from, villages were cited by more than half the responding programmes (12) as an obstacle, followed by lack of funding (11), and/or available supervisors or coordinators (6); 5 programmes also cited COVID-19 related restrictions.

#### Programme funding and malaria CHW compensation

**Fig 4:**
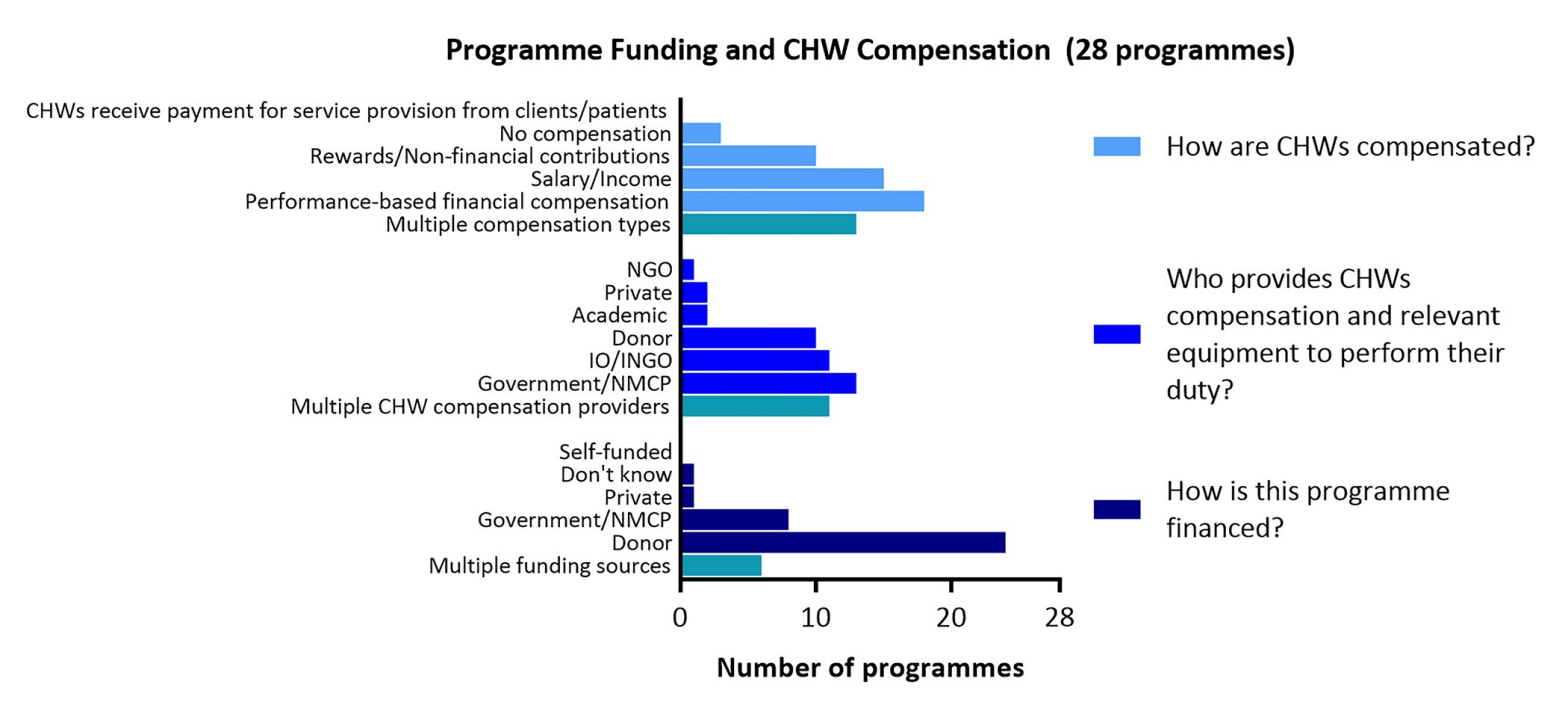
Programme funding and CHW compensation.

**Fig 4** shows sources and types of programme funding and malaria CHW compensation. Twenty-four programmes (out of 28) were financed through donor funding; 18 donor funding alone and 6 in combination with government funding. Only 2 programmes were fully funded by their respective governments (IND2 and VNM2), and 1 fully funded by a private entity (IND1). Financial sources for CHW compensation and equipment supply were more diverse, with responses citing government and/or NMCPs as a provider for 13 programmes, followed by IOs/INGOs (11), and donors (10). A larger number of programmes relied on a variety of funding sources to provide CHW incentives or compensation (11) than overall programme funding (6). Thirteen programmes provided CHWs with a combination of compensation types. The most common type of CHW compensation was financial incentives based on performance (18), followed by monthly salary (15), non-financial incentives or rewards (10), or no compensation for volunteer CHWs in BTN1, LKA1, and MMR7. None of the CHWs were compensated through their service provision, and subsequently no responses reported self-funded programmes.

Eighteen programmes reportedly partnered with other organisations to implement the CHW programmes to provide training (15), supervision (15), and materials or supplies for CHWs to perform their roles (11).

#### Programme evaluation

**Fig 5:**
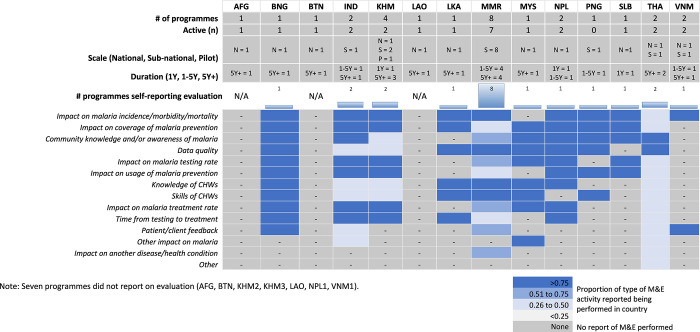
What kind of evaluation is being performed in each country?.

Twenty-one programmes reported performing programme evaluations: either by their own organisation (14), an external party (5), or both (BNG and MMR5). Across countries, the most commonly evaluated dimensions were: impact on malaria incidence, morbidity or mortality (18 programmes in 10 countries); followed by coverage of malaria prevention (14), community knowledge and/or awareness of malaria (15), and data quality (16). Programmes also evaluated malaria CHWs’ knowledge (15) and skill (15), malaria testing rate (15), malaria treatment rate (14), usage of malaria prevention (12), and time from testing to treatment (11). Nine programmes reported assessing feedback from their patients or clients. Only 2 programmes reported evaluating programme impact on other diseases beyond malaria (MMR6 and THA1). **Fig 5** summarizes the 13 reported evaluation dimensions at the country level, including the frequency of evaluation topics.

### Programme implementation

#### Implementation during the COVID-19 pandemic

Seventeen programmes reported malaria CHWs performing additional roles during the COVID-19 pandemic. In BNG and MMR2, malaria CHWs organised COVID-19 awareness campaigns, promoted vaccination, and informed communities about the use of personal protection equipment. Malaria CHWs were tasked with fever surveillance and migration tracking in 5 programmes, particularly screening for suspected cases by taking temperature and tracing contacts (IND and AFG), through household visits (BNG) or at a quarantine centre or health facility (LKA). In Nepal, a plan to assign Malaria Village Workers to perform integrated testing for COVID-19, tuberculosis, and HIV was described.

Twenty-one programmes described experiencing implementation challenges during the pandemic, mainly due to travel and activity restrictions. Sixteen programmes reported reduction, delay, or suspension of training and supervision activities resulting in less frequent visits or meetings between malaria CHWs and their supervisors. Due to travel restrictions, three programmes described limitations with providing services such as indoor residual spraying in the community (LKA), mass gathering for health education sessions (MMR5), and patient referral for ICMV-related diseases, namely dengue, lymphatic filariasis, tuberculosis, HIV/AIDS and leprosy [14].

Survey responses also described increased workload and limited time for malaria CHWs to perform their normal routine work, such as immunizations and dengue case investigation, during the pandemic. Shortage of staff at health facilities (IND1), resources and commodities (AFG and LAO), and funding (LKA and PNG) were also reported as challenges. Seven programmes described minimal impact or changes during the pandemic.

To cope with the limitations, programmes reported shifting their activities online. Where internet connection was available, activities were conducted virtually using mobile applications. In-person training groups were reduced in size to minimize mass gatherings, however face-to-face supervisory meetings were only conducted when traveling was allowed. In Bangladesh, phone calls ensured treatment compliance for malaria, and for COVID-19 related services, including informing patients about COVID-19 measures such as mask usage, hand washing, and social distancing. MMR2 reported that the integrated community case management programme (iCCM or the integrated management of malnutrition, pneumonia, diarrhoea, and acute respiratory infections for children under 5 years) experienced minimal impact from COVID-19 due to the community-based delivery model which helped ensure continuation of the health services provided by malaria CHWs.

#### Views on programme sustainability

**Fig 6:**
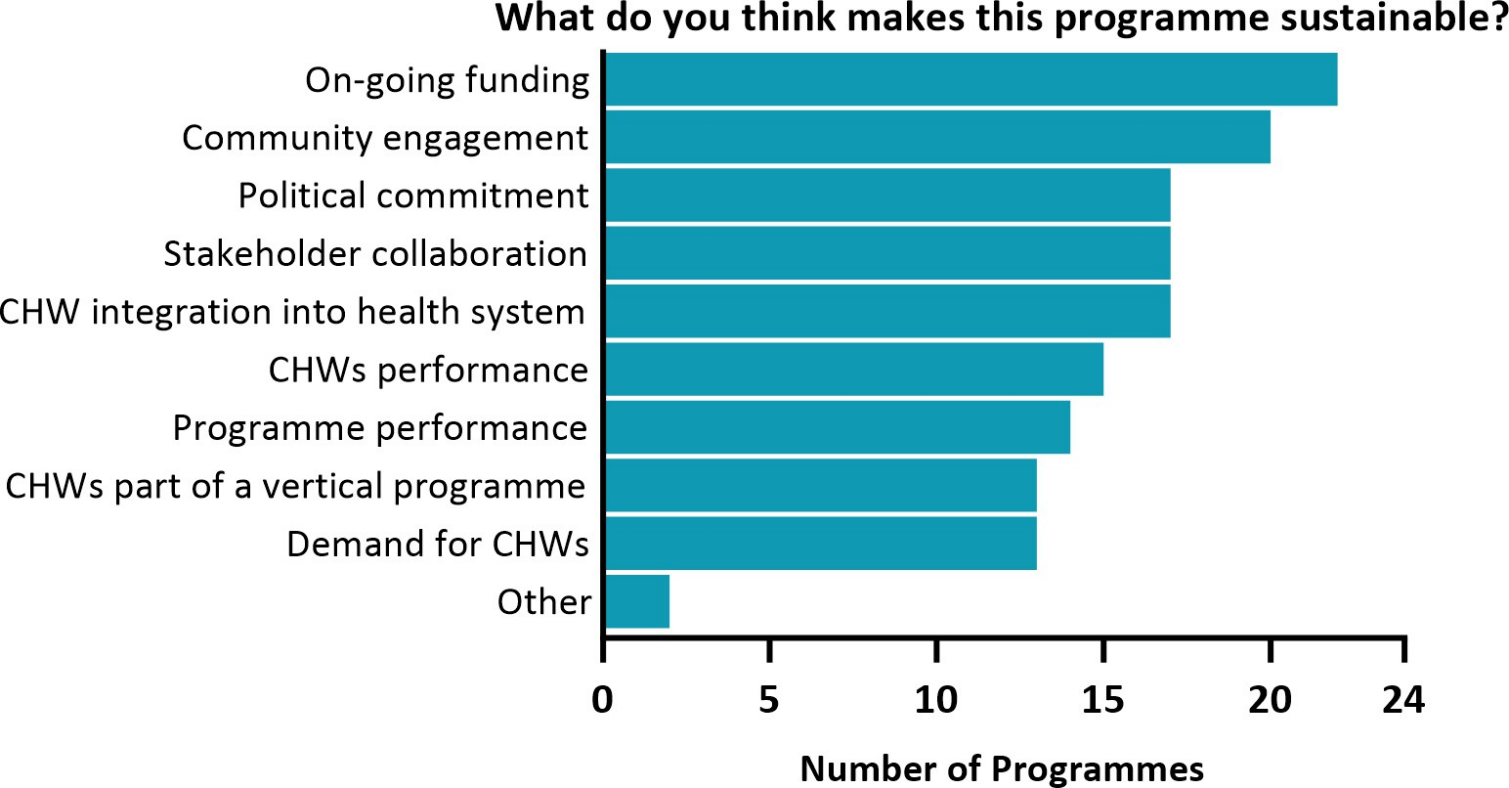
What do you think makes this programme sustainable?.

**Fig 6** summarizes self-reported views of 24 active programmes, for which respondents may select multiple factors that contribute to sustainability. Ongoing funding (22 programmes), community engagement (20), political commitment (17), and stakeholder collaboration (17) were reported as crucial to programme continuation. Ensuring the performance of malaria CHWs (15) and the programmes themselves (14) were also perceived as contributing to programme sustainability. Echoing reported enabling factors such as expanding malaria CHW roles by integration (17) and on-going demand for malaria CHWs (13), a programme respondent in Myanmar indicated that "the fact that the [malaria] CHWs provided a broader package of health activities made them effective and sustainable. Patients visit because they are ill, not necessarily due to malaria. If you want to have a good uptake, you need to address most health issues of the patient”. Thirteen programmes also reported that being part of a vertical programme sustains malaria CHWs’ work in providing ongoing services. Additionally, AFG reported that integration of malaria CHWs’ roles into training curricula, regular research-based evaluations, and partnership between public and private sectors, were enabling factors for sustaining their programme. All 4 inactive programmes reported lack of funding as the reason for discontinuation.

## Discussion

Across Asia-Pacific countries, health promotion and education for malaria and other diseases are the most frequently reported services provided by malaria CHWs, as also highlighted in a recent global article series [15]. Besides COVID-19 related activities and referral services, other common non-malaria services reportedly provided by malaria CHWs include services related to reproductive and maternal health care, family planning, new-born and child health, malnutrition, other communicable diseases and diarrhoea. This largely reflects the traditional focus of CHW programmes which were designed to target maternal, new-born and child health interventions in lower- and middle-income countries (LMICs) [15–17].

As 12 programmes in 8 countries reported malaria CHWs providing non-communicable diseases (NCDs) services, our findings also support the emerging role of malaria CHWs in addressing/preventing NCDs, with past literature suggesting the potential to package these roles with health promotion and educational activities to strengthen NCD programmes [18]. Examples of malaria CHWs being able to effectively address NCDs were reported beyond these 12 programmes. Trained Behvarz CHWs in Iran were reported to be effective at controlling hypertension and diabetes [19], and health promotion delivered as a package by lady health workers in Pakistan was found to reduce the onset of hypertension in children and young adults [19]. Unfortunately, this survey did not receive any responses from Iran and Pakistan, both of which were targeted programmes identified from this project’s systematic review [11].

In the GMS context, VMWs have taken on the role of health promoters sustaining malaria awareness within their communities, and may be further tasked to observe the health needs of their community and address other health concerns based on the community’s feedback [20]. Several operational research studies on the expansion of malaria CHW roles have already been tested in the subregion. In western Cambodia, VMWs take on this broader preventive role through the integration of health education packages on hygiene and sanitation, disease surveillance and first aid, management of mild illness, and vaccination and antenatal care. They have also been tasked to screen febrile patients using multiplex RDTs to detect dengue anti-bodies [21]. Feasibility studies into this strategy suggests that deploying user-friendly point-of-care tests selected for local pathogens, along with disease-specific education that is tailored to the literacy level and limited resources of VMWs, could be feasible and acceptable [22, 23]. Additionally, in Myanmar, where malaria CHW roles have been expanded to include a basic healthcare package that incorporates the treatment of malnutrition, diarrhea, and respiratory tract infections, observational studies have found that this integration did not significantly impact the uptake of malaria services [24] or even contributed to sustaining malaria service uptake [25]. These examples demonstrate how malaria CHWs can take on a broader preventive role and tailor their efforts to fit the health needs of the community, without compromising their original mandate of conducting malaria surveillance and delivering malaria services.

Our findings show that several groups of malaria workers were tasked to perform COVID-19 activities in addition to providing their original service package. Globally, there were concerns that the pandemic will reduce donations from developed countries, thus hindering elimination efforts, especially in Africa [26], and contribute to re-establishment of malaria in countries which have recently eliminated it [27]. African countries have emphasized the need to maintain malaria control interventions alongside the pandemic mitigation strategies [25]. WHO has recommended that tailoring interventions, such as encouraging early care-seeking for fever and ensuring diagnostic confirmation for both malaria and COVID-19 in endemic communities, can help maintain provision of essential services during times of competing priorities [26]. CHWs have been recognized for contributing to COVID-19 vaccination plans by overcoming access and equity barriers in LMICs [28]. This suggests that malaria CHW programmes conducting routine epidemiological surveillance provide the readily available human resources and logistics that may be leveraged to respond to public health emergencies and lessen the burden on health centres. Furthermore, our findings show that many programmes have adapted programme activities to maintain functionality during the pandemic, such as integrating phone-based services to ensure compliance for malaria and COVID-19 services and shifting training sessions online when feasible. Operationalizing online and digital tools help malaria CHWs and programme staff maintain programme functionality despite the restrictions during the pandemic. It also shows prospects to quickly adapting malaria CHWs to take on new roles in supporting future epidemic control responses [29]. Understanding the response to COVID-19 therefore remains relevant, as it provides valuable lessons for future public health interventions leveraging community-based health workers, particularly in remote and resource-limited settings.

In the GMS, where donor funding is the main source of financing for the largely vertical malaria programmes, sustaining funding for malaria CHWs or VMWs is vital. Our findings found most programmes identified in this survey were chiefly funded by donors, with sub-recipients such as NMCPs and NGOs in each country providing compensation for malaria CHW services under their management. Although not evident in this survey, it should be noted that different CHW cadres may receive different types of compensation. For example, while facility-based government health workers such as the Behvarz workers in Iran or Auxiliary Nurse-Midwives (ANM) in India, generally receive a monthly salary, it is more common that volunteer workers only receive travel allowances and/or performance-based incentives [30]. The payment discrepancy between different cadres of workers have been reported to demotivate volunteers, such as is reported by the Village Malaria Workers in Cambodia [31]. Financial incentives have been cited elsewhere as crucial motivators for CHWs in general [3] and their removal may result in reduced provision and quality of services, and higher attrition rates [32]. Reflections from trialling expanded CHW service packages in Cambodia [23] and Myanmar [33, 34] recommended that workers should receive higher cash incentives to match the increasing workload when performing integrated roles. As such, at the CHW level, considerations for expanding their role entails continuing to provide financial support or incentives, such as performance-based incentives, or introducing the aforementioned remuneration schemes to those not receiving monthly compensation.

At the programme level, the importance of long-term investment for malaria elimination programmes [35] and continuity of financial support are emphasized elsewhere [36, 37]. Experiences of countries that established linkages between CHWs and local health facilities through additional financial support from public health funding offer some important lessons on how to formalize CHW integration into the national public health system. These examples were found in public health programmes in Afghanistan, India, and Sri Lanka, or well-established NGOs such as BRAC in Bangladesh. Past experiences also show that malaria programmes benefited from integrating into primary health care systems by delegating malaria activities to well-established local cadres of community workers, such as Nepal’s Female Community Health Volunteers (FCHVs) [38, 39] and Sabah’s Primary Health Care Volunteers (PHCV) in Malaysia [40, 41]. How different programmes may approach integration will depend on the context and enabling factors which require in-depth understanding of how programmes are established, managed, and implemented. The various approaches for integration are further explored in this project’s subsequent interviews with implementers which are reported elsewhere.

Investigation into cost effectiveness of CHW programmes or a comparison of CHW programme expenditure against other measure for improving population health will be needed to compel both donors and domestic governments to invest in strengthening an integrated primary health care delivery system [42]. Researchers have argued that efforts to strengthen primary health care systems and fund CHW programmes are often distorted by various factors including donor’s focus on funding disease-specific vertical programmes and domestic government’s disproportionate focus on funding specialist, tertiary care [37, 43]. As such, malaria CHW programmes may need to document evidences showing the positive impact on both malaria and non-malaria outcomes using tailored set of indicators, in order to show the potential yields of investing in malaria CHW programmes on strengthening the overall primary health care delivery systems, which then contribute to sustaining malaria services as well. Simultaneously, other approaches may also be explored such as considering how CHW programmes may be funded and supported through partnerships between local government units and private entities. This survey identified programmes in Papua New Guinea [44] and India [13], whereby partnerships between local government and a private entity was leveraged to fund and implement malaria CHW programmes. However, from survey findings, it is unclear whether these implementation approaches are sustainable or not.

Evaluation of impact on malaria incidence, morbidity, or mortality has been primarily reported across identified programmes. However, these assessments may be reported internally and thus not previously identified in the literature and omitted from this project’s systematic review [11]. This survey identified only 2 programmes that evaluated their impact on diseases beyond malaria. For GMS countries, showcasing the positive impacts of expanded malaria CHW programs on various disease outcomes and on their performance may help leverage ongoing funding for these programmes. Programmes need to consider how additional roles may reduce malaria CHW productivity and service quality, as malaria CHWs are faced with competing tasks leading them to perform one role over the other based on feasibility, renumeration, and/or preferences [45–47]. Past systematic reviews described roles and clusters of roles performed by CHWs [3, 48] and those commonly performed alongside malaria services across countries [49], but not as many have reviewed the impacts these roles have on targeted health outcomes. Fundamentally, our findings demonstrate the resilience of malaria CHWs through COVID-19 and their potential to perform a myriad of roles alongside malaria services across the Asia Pacific. Future research should focus on measuring the impact of additional roles on CHW performance and various disease outcomes to verify the benefits and feasibility of expanding different roles.

### Strengths and limitations

The study benefitted significantly from the preliminary findings of this project’s systematic review, and piloting of survey questions with a focus group of implementers during the survey development. For example, focus group feedback allowed appropriate revision of the survey to capture multiple types of CHW within each programme who may provide similar or different services. The survey adds to the findings of the systematic review by implementing the same set of questions for all programme respondents, thus allowing comparability across programmes in the analysis. This addresses some of the potential risks of bias that are inherent in systematic reviews. Additionally, the survey collected self-reported anonymous data which allowed for less social desirability bias.

Free text responses in the survey elicited more in-depth information on the perceived reasons, changes, and challenges of the programme implementation, particularly regarding their sustainability and impact of COVID-19. The survey was distributed among APMEN partners and wider networks to ensure coverage of malaria programme implementers across the Asia Pacific. Due to the voluntary nature of the survey, we did not receive responses from all countries in the region, and received multiple responses from countries with multiple malaria CHW programmes and/or that actively engage with APMEN, such as the CSOs in Myanmar. Additionally, as many programmes are sub-recipients with direct ties to donors, they may be more motivated to respond and face potential bias in responses given their reliance on donor funding.

This survey excludes programs that manage CHWs exclusively providing malaria services; therefore, the findings do not document prospective expanded roles or other programmatic factors that may ensure sustainability from the perspectives of programs that have not yet expand the roles of their CHWs. As the survey allowed for multiple responses from each implementing organisation, inconsistent data reported by respondents within the same programmes and other reconciliations on critical data points were rechecked with a representative of the responding organisation. It is also likely that some information is unknown to the respondents and thus may not be specified in their responses; for example, specific services provided by the malaria CHWs or evaluation aspects of the programmes. Therefore, absence of service provision or other programme characteristics reported for each country in this survey should be interpreted as missing data, rather than lack thereof. Additionally, the survey collects and reports on challenges experienced by CHW programmes during the COVID-19 pandemic. While this was a prominent concern when the survey was developed and implemented, many of the challenges experienced during this period may no longer be relevant.

## Conclusion

This scoping survey identified 28 programmes in 14 countries in the Asia Pacific region where CHWs provide health services beyond malaria. Our findings confirm that malaria CHWs provide a myriad of services in addition to malaria, although these are primarily traditional CHW roles in health education and promotion, and maternal and child health. The biggest challenge faced in many programmes was lack of, or declining, donor funding. Therefore, central to the discussion on sustaining malaria elimination efforts in the GMS region is determining how to source ongoing funding for malaria programmes by integrating with other vertical programmes or into existing primary health care programmes. Adaptability of malaria CHW programmes in the region by integrating COVID-19 related activities and emergency response efforts with their malaria duties demonstrates the resilience of CHWs under changing public health landscapes. For the GMS, more research is needed to contribute to the existing body of evidence measuring the impact of additional roles on malaria CHW performance and various disease outcomes to assess the feasibility of expanding certain roles to correspond with the specific health needs of the communities they serve. Strategies for role expansion and integration have been investigated in subsequent case studies and feasibility studies in the Asia Pacific as part of this project, which will inform an implementation package for expanding malaria CHW roles in GMS countries.

## Supporting information

S1 Appendix Survey on expanded roles of malaria community health workers in the Asia-Pacific. (DOCX)

S2 Appendix Baseline characteristics of excluded survey respondents and description of ‘other’ responses selected in the survey. (DOCX)
