# Supplementary material for: Expanded roles of community health workers to sustain malaria services in the Asia-Pacific: A landscaping survey
Source: PLOS Glob Public Health. 2024 Aug 14;4(8):e0003597. doi: 10.1371/journal.pgph.0003597 (PMC11324099; doi:10.1371/journal.pgph.0003597)
Supplement: S1 Appendix — (DOCX) [file pgph.0003597.s001.docx]

**S1 Appendix. Survey on expanded roles of malaria community health workers in the Asia-Pacific**

Survey URL: <https://oxford.onlinesurveys.ac.uk/survey-on-expanded-roles-of-malaria-chws-in-the-asia-pacific> [launched 30 September 2021]

[Log-in page]

Please access the survey using the password in the invitation email.

If you have any questions or trouble accessing this survey, please contact:

- Massaya Sirimatayanant [[massaya@tropmedres.ac](mailto:massaya@tropmedres.ac)]
- Monnaphat Jongdeepaisal [[monnaphat@tropmedres.ac](mailto:onnaphat@tropmedres.ac)]

Survey password: [MORU2021]

**P. 1 Introduction**

***General Information***

This survey is being conducted by Mahidol-Oxford Tropical Medicine Research Unit in collaboration with the Asia-Pacific Malaria Elimination Network. This study comprises of this **scoping survey of community health worker (CHW) programmes** followed by **interviews with implementers of selected programmes**. We aim to answer the following questions: (1) which organisations provide village malaria worker or equivalent services in the Asia-Pacific; (2) what expanded roles have been used (if any) and which have been rolled out beyond initial pilots; (3) what strategies were used to sustain the programmes and what evidence is there for impact. **“Malaria CHWs”** in this study are any type of community health worker, village health worker, village malaria worker or health volunteer who provides any services for malaria in a community setting. **“Expanded roles”** refers to any services these same health workers provide for a health condition other than malaria.

This survey aims to identify implementations of programs in which CHWs provide health services for malaria alongside other health services in the Asia-Pacific region. The evidence generated will be used to inform efforts to sustain VMW networks in the Greater Mekong Subregion. You have been invited to participate in this survey because of your organizations’ affiliation with the Asia-Pacific Malaria Elimination Network (APMEN) and/or its involvement in managing VMWs in this region.

Malaria CHWs have played an important role in providing diagnosis and treatment of malaria in remote/rural areas. They may be established as a key component of a malaria elimination programme externally sponsored by a donor or as an extension of national public health programmes through expanding the roles of CHWs to include malaria services.

This survey is a part of the landscaping analysis for a research priority on VMWs under the Regional Artemisinin-Resistance Initiative (RAI3E). Programmes identified in this landscaping survey that fit the research criteria will be selected for further interview by a team of researchers from our partner organization, the Global Health Group, University of California, San Francisco (UCSF). Representatives of the selected programs will then be contacted and asked to voluntarily participate in implementer interviews to explore more about your programmes and organisations.

***Participation***

Participation in this survey is completely voluntary. If you decide to participate, you may withdraw at any point for any reason before submitting your answer by closing the browser. If you wish to withdraw from the study after the submission or completion of the survey you can contact the research team.

***Data usage***

We will only ask you to identify the name of the organization/country which you are responding on behalf of. We will not collect any data that could directly identify you. Your IP address will not be stored. The research team will maintain the confidentiality of the research records or data, all data will be stored in an encrypted and secure database at MORU and stored for a minimum of 10 years after we publish the results. As this survey is being conducted on a third-party website, the research data may be stored on backups or server logs beyond the timeframe of this research project (1 August 2021 – 30 June 2022). Although every reasonable effort has been taken, confidentiality during communication via the internet cannot be guaranteed.

***Data Access***

The research team at MORU is the data controller, and will determine how your response data is used in the study. We will process the data you provide for the purposes of the research outlined above.

Your data may be shared with other investigators taking part in this study, the APMEN Secretariat and a team of researchers at the Global Health Group, UCSF Institute for Global Health Sciences.

***Study Review***

This research project has been reviewed by, and received ethics clearance, from the University of Oxford Tropical University Research Ethics Committee [534-21].

***Who do I contact if I have a concern or I wish to complain?***

If you have a concern about any aspect of this study, please contact

- Massaya Sirimatayanant [[massaya@tropmedres.ac](mailto:massaya@tropmedres.ac)]
- Monnaphat Jongdeepaisal [m[onnaphat@tropmedres.ac](mailto:onnaphat@tropmedres.ac)]

We will do our best to answer your query. We will acknowledge your concern within 10 working days and give you an indication of how it will be dealt with. If you remain unhappy or wish to make a formal complaint, please contact the Chair of the Research Ethics Committee at the University of Oxford who will seek to resolve the matter as soon as possible:

- The Chair, Oxford Tropical Research Ethics Committee;

Email: oxtrec@admin.ox.ac.uk; Address: Research Services, University of Oxford, Wellington Square, Oxford OX1 2JD

This survey is composed of about 30 questions. Participation will take about 15 – 20 minutes.

**Please note that you may only participate in this survey if you are 18 years of age or over.**

1. Are you 18 years or above?
   1. Yes
   2. No

**P.2 Consent**

**I have read the information above and I agree to participate in the understanding that the data (any information you provide about your organisation/country’s malaria CHW programme) I submit will be processed accordingly.**

1. I consent to participate in this survey
   1. Yes
   2. No / *redirect to screen out message*

[Screen out message]

You have been screened out of the survey either because you are below 18 years old and/or did not give your consent to participate in this survey.

Please contact Massaya Sirimatayanant ([massaya@tropmedres.ac](mailto:massaya@tropmedres.ac)) or Monnaphat Jongdeepaisal ([monnaphat@tropmedres.ac](mailto:monnaphat@tropmedres.ac)) if you have further questions, concerns or would like to provide feedback about the survey or the RAI3E VMW research project.

**P.3 About your organisation**

1. What type of organization do you work for?
   1. Government/National Malaria Programme
   2. Academic/Research institution
   3. NGO/INGO
   4. Donor
   5. Private sector
   6. International organisations (e.g. UN, WHO)
   7. Other, please specify.
2. In which Asia-Pacific countries does your organisation work? Select all that apply.
   1. Afghanistan
   2. Bangladesh
   3. Bhutan
   4. Cambodia
   5. China
   6. DPR Korea
   7. India
   8. Indonesia
   9. Lao PDR
   10. Malaysia
   11. Myanmar
   12. Nepal
   13. Pakistan
   14. Papua New Guinea
   15. Philippines
   16. Republic of Korea
   17. Solomon Islands
   18. Sri Lanka
   19. Thailand
   20. Timor-Leste
   21. Vanuatu
   22. Viet Nam
   23. Other, please specify.
3. What is the name of your organisation?
4. What is your position/job designation?
5. Does your organisation have any programmes that manage CHWs who provide malaria services?
   1. Yes
   2. No / *skip to P.10 Recommendations*

Note: Please note that **malaria CHWs in this survey refer to any type** of community health worker, village health worker, village malaria worker or health volunteer **who provides any services for malaria in a community setting.**

**P.4 About your organisation (2)**

1. Do these CHWs who provide malaria services **also provide other health services**?
   1. Yes
   2. No / *skip to P.10 Recommendations*

**P.5 About your organisation (3)**

1. Can you provide more detail about this programme?
   1. Yes
   2. No / *skip to P.10 Recommendations*

Note: If you wish for your co-worker(s) to fill in the responses for this programme(s), you are welcome to forward the invitation email to them.

**P.6 About CHWs and their roles**

**Note:** We are interested to know more about the **CHWs who provide malaria as well as other services in your programme.**

- Please respond to the following questions to reflect what is happening right now in the programme.
- Please **type in or select 'Don't know'** to questions that you do not know the response for.

1. What is the name of this programme?
2. In which Asia-Pacific country (or countries) does it operate?

*(Please note that our definition of programme is country specific; if your programme operates in multiple countries, you may choose to respond individually about the programme in each respective country. The survey will allow you to respond about another programme after you have completed to this set of questions about the programme in the first country.)*

- 1. (List of countries as in no.5)
  2. Don’t know

1. Who manages the programme? Select all that apply.
2. Government/National Malaria Programme
3. Academic/Research institution
4. NGO/INGO
5. Donor
6. Private sector
7. International organisations (e.g. UN, WHO)
8. Don’t know
9. Other, please specify.
10. Is there **more than one type of malaria CHW with different sets of roles** who work with this programme? (*If you select “yes” you will be shown a set of questions for each type of CHW*)
11. Yes
    - 1. What is the name of the **first** type of malaria CHWs?
      2. What **malaria service(s)** do CHWs provide in this programme? Select all that apply.
    - Testing with rapid diagnostic test (RDT)
    - Testing with microscopy
    - Treatment with antimalarials
    - Vector control activities e.g. distribution of mosquito nets
    - Referral of malaria patients to health facilities
    - Health promotion/education for malaria
    - Don’t know
    - Others, please specify.
      1. What **non-malaria service(s)** do CHWs provide in this programme? Select all that apply.
    - Referral of patients to health facilities
    - Health promotion/education for other disease/topics
    - Non-malarial febrile illness diagnosis
    - Non-malarial febrile illness treatment
    - Activities related to tuberculosis
    - Activities related to pneumonia
    - Activities related to diarrhoea
    - Activities related to Covid-19
    - Activities relating to specific infectious diseases
    - Family planning, child health services, maternal health care
    - Activities related to nutrition and/or malnutrition
    - Activities related to the treatment of other non-communicable disease
    - Health services for bed-ridden patients
    - Substance abuse
    - Mental health services
    - Don’t know
    - Others, please specify.
      1. Have any of these roles changed due to COVID-19, if so how?
      2. What is the name of the **second** type of malaria CHWs?
      3. What **malaria service(s)** do CHWs provide in this programme? Select all that apply.
    - (list of services)
      1. What **non-malaria service(s)** do CHWs provide in this programme? Select all that apply.
    - (list of services)
      1. Have any of these roles changed due to COVID-19, if so how?
      2. Is there a **third** type of CHWs that provide malaria and other services?
    - Yes
      - (repeat sub-questions i - v)
      - If there are **more than 3** types of CHWs that provide **malaria and other services**, please give their names and briefly describe their roles in the space below?
    - No
12. No
13. What is the name of the malaria CHWs?
14. What **malaria service(s**) do CHWs provide in this programme? Select all that apply.
    - (list of services)
15. What **non-malaria service(s)** do CHWs provide in this programme? Select all that apply.
    - (list of services)
16. Have any of these roles changed due to COVID-19, if so how?

**P.7 Programme-specific questions**

**Note:** We are interested to know more about how the CHWs are managed.

- Please respond to the following questions to reflect what is happening right now in the programme.
- Please **type in or select 'Don't know'** to questions that you do not know the response for.

1. How are the CHWs selected? Select all that apply.
2. Elected by the community
3. Recruited by the managing organisation
4. Don’t know
5. Other, please specify.
6. Do CHWs receive training for their service provision?
7. Yes
8. For which service(s)? Select all that apply.
   - (List of services in no.13)
   - Don’t know
9. What kind of training? Select all that apply.
   - Introductory training
   - On-the-job training
   - Refresher training in groups
   - Refresher training one-on-one
   - Online training
   - Don’t know
   - Other, please specify.
10. How often do they receive training?
    - Monthly
    - Quarterly
    - Biannually
    - Annually
    - Don’t know
    - Other, please specify.
11. From whom do the CHWs receive training? Select all that apply.
    - Government/National Malaria Programme
    - Academic/Research institution
    - NGO/INGO
    - Donor
    - Private sector
    - International organisations (e.g. UN)
    - Don’t know
    - Other, please specify.
12. No
13. What are the factors, if any, that limit the provision of training? Select all that apply
14. None
15. Lack of funding
16. Lack of available trainers
17. Lack of updated training materials (e.g. guidelines)
18. Lack of interest from CHWs
19. Don’t know
20. Other, please specify.
21. Has CHW training changed due to COVID-19, of so how?
22. Do CHWs receive supervision?
23. Yes
24. Where do CHWs meet with their supervisors? Select all that apply.
    - At the community where CHWs work
    - At nearest health facility
    - Don’t know
    - Others, please specify.
25. How often do CHWs meet with programme supervisors/coordinators?
    - Monthly
    - Quarterly
    - Biannually
    - Annually
    - Don’t know
    - Other, please specify.
26. Who supervises/coordinates the CHWs? Select all that apply.
    - Government/National Malaria Programme
    - Academic/Research institution
    - NGO/INGO
    - Donor
    - Private sector
    - International organisations (e.g. UN)
    - Don’t know
    - Other, please specify.
27. No
28. What are the factors, if any, that limit the provision of supervision? Select all that apply.
29. None
30. Lack of funding
31. Lack of access to/from village
32. Lack of available supervisor/coordinator
33. Lack of interest from CHWs
34. Don’t know
35. Other, please specify.
36. Has CHW supervision changed due to COVID-19, if so how?
37. Has your organization partnered with other organizations to implement this programme?
38. Yes
39. What roles do these partner organizations perform? Select all that apply.

- Provision of funding
- Provision of training
- Provision of supervision
- Provision of materials or supplies
- Don’t know
- Other, please specify.

1. Which organisation(s)?
2. No
3. How is this programme financed? Select all that apply
4. Self-funded through payment from patients to CHWs
5. Government funding
6. Donor funding
7. Don’t know
8. Other, please specify.
9. How are the CHWs compensated? Select all that apply
10. CHWs receive payment for service provision from their clients/patients
11. Monthly salary or income
12. Financial incentives based on performance (e.g. incentive per number of RDT done)
13. Rewards or other non-financial contributions
14. No compensation
15. Don’t know
16. Others, please specify.
17. Who provides CHWs compensation and relevant equipment to perform their duty? Select all that apply.
18. Government/National Malaria Programme
19. Academic/Research institution
20. NGO/INGO
21. Donor
22. Private sector
23. International organisations (e.g. UN, WHO)
24. Don’t know
25. Other, please specify.
26. For how long has this programme been implemented?
27. Less than 6 months
28. 6 months to less than 1 year
29. 1 to 5 years
30. More than 5 years
31. Don’t know
32. How many CHWs are currently involved in the programme*?*
33. How many health facilities/health posts are involved in the programme?
34. Is the programme evaluated for its implementation?
35. Yes
36. On which dimensions is the programme evaluated? Select all that apply.

- Impact on malaria incidence
- Impact on malaria testing
- Impact on malaria treatment
- Impact on malaria prevention (e.g. use of mosquito nets)
- Community knowledge and/or awareness of malaria
- Other impact on malaria
- Impact on another disease/health condition
- Data quality
- Patient/client feedback
- Knowledge of CHWs
- Skills of CHWs
- Don’t know
- Other, please specify.

1. Who evaluates the programme? Select all that apply.

- By your organization
- By an external organization
- Don’t know
- Other, please specify.

1. Could you share where we could read more about it if the evaluation is publicly available? (e.g. URL of website, published articles, programme report)
2. No
3. Don’t know
4. Other, please specify.
5. Is this programme still active?
6. Yes
7. What do you think makes this program sustainable? Select all the apply.

- Ongoing funding
- Political commitment
- Community engagement
- Stakeholder collaboration
- CHWs performance (e.g. quality of service)
- Demand for CHWs (e.g. ongoing uptake of the services)
- Don’t know
- Other, please specify.

1. No
2. Why is this programme no longer active? Select all that apply.

- Lack of funding
- Lack of political commitment
- Lack of community engagement
- Lack of stakeholder collaboration
- High attrition rate of CHWs (i.e. CHWs leave the programme)
- Replaced by another programme
- Programme no longer relevant (e.g. lack of malaria cases or community interest in the services)
- Don’t know
- Other, please specify.

1. Has CHW programme been impacted in any other ways than services provided, supervision or training by the COVID-19 pandemic (e.g. funding, monitoring and evaluation, partnership, etc.)?
2. Yes
3. How? Please describe.
4. No

Note: **Thank you for your responses about this programme.**

Following the completion of this survey, the study team will be conducting interviews with implementers of selected programmes. We are interested in interviewing individuals who are involved in your organisation at the managerial level and able to provide an overview of the implementation process of the programme.

1. Could you please identify an individual(s) in your organization we should invite to be interviewed further about the programme? (i.e. name and contact information)

Note: If you would like describe **another** programme in your organisation that work with CHWs, please select **'Yes’** in the question below.

1. Can you identify **another specific programme implemented by your organisation** in which malaria CHWs provide other health services in addition to those for malaria?
2. Yes / *repeat program-specific questions (up to additional 2 times)*
3. No / *go to P.15 Recommendations*

**Note: Please note that if you select yes, you will be asked to fill out a set of responses for each programme that you identify.**

You will able to fill in the responses for up to 3 programmes in total.

If you wish for your co-worker(s) to fill in the responses for another programmes, you are welcome to forward the invitation email to them.

**P. 10 Recommendations**

1. Can you recommend other organizations that have CHWs providing malaria and/or other health services in the Asia-Pacific region? Please specify the name of the programme(s) as well if you know.

**P. 11 Final page**

Thank you for taking the time to complete this survey. Your responses will be analysed by the research team; if your program fits the study criteria we may contact your organization to request setting up a voluntary implementer interview soon.

Please contact Massaya Sirimatayanant ([massaya@tropmedres.ac](mailto:massaya@tropmedres.ac)) or Monnaphat Jongdeepaisal ([monnaphat@tropmedres.ac](mailto:monnaphat@tropmedres.ac)) if you have further questions, concerns or would like to provide feedback about the survey or the RAI3E VMW research project.

**--- End of Survey ---**
