# Supplementary material for: Expanded roles of community health workers to sustain malaria services in the Asia-Pacific: A landscaping survey
Source: PLOS Glob Public Health. 2024 Aug 14;4(8):e0003597. doi: 10.1371/journal.pgph.0003597 (PMC11324099; doi:10.1371/journal.pgph.0003597)
Supplement: S2 Appendix — (DOCX) [file pgph.0003597.s002.docx]

**S2 Appendix. Baseline characteristics of excluded survey respondents and description of ‘other’ responses selected in the survey**

**Baseline characteristics of** **ineligible survey respondents (n=19)**

|  | Respondents | |
| --- | --- | --- |
| Country |  |  |
|  | n | % |
| Afghanistan | 1 | 5.3 |
| Bangladesh | 1 | 5.3 |
| Bhutan | 1 | 5.3 |
| Iran | 1 | 5.3 |
| Myanmar | 2 | 10.5 |
| Papua New Guinea | 1 | 5.3 |
| Philippines | 1 | 5.3 |
| Solomon Islands | 1 | 5.3 |
| Thailand | 1 | 5.3 |
| Viet Nam | 3 | 15.8 |
| Multiple countries | 6 | 31.6 |
| Organisation type |  |  |
| Academic/Research Institution | 2 | 10.5 |
| IO/INGO | 5 | 26.3 |
| NGO | 9 | 47.4 |
| Government/NMCP | 2 | 10.5 |
| Donor | 1 | 5.3 |
| Private sector | 0 | 0.0 |
| Work position |  |  |
| Director/manager | 8 | 42.1 |
| Project/programme officer | 4 | 21.1 |
| Technical advisor/Specialist | 7 | 36.8 |

**Description of ‘other’ responses selected in the survey**

| Category | Programme | CHW cadre(S) | ‘Other’ free text response |
| --- | --- | --- | --- |
| Malaria services provided by CHWs | IND1 | Malaria Field Coordinators | Supervision and supply chain management of commodities for Village Malaria Workers |
|  | LKA | Public Health Inspectors | Training of newly appointed Public Health Inspectors (refresher courses) |
|  | MMR6 | Community Health Workers/ Integrated Community Health Worker | Mass screening and treatment for high transmission areas |
|  | VNM1 | Village Health Worker | Follow up patients to ensure treatment adherence with antimalarial |
| Non-malaria services provided by CHWs | IND1 | Malaria Field Coordinators | Training of Government Health Staff towards other health programmes |
|  | LKA | Public Health field officer | Other mosquito-borne diseases - dengue, filariasis etc. |
|  |  |  |  |
| Training Frequency | AFG | N/A | Depends on the report and recommendation of CHWs supervisors, annual plan of the health services provision contracted NGOs and availability of funding |
|  | MMR5 | N/A | In addition to monthly, quarterly and annual, training is also provided when needed |
| Training Provider | THA1 | N/A | Community based organisations |
|  |  |  |  |
| Supervision Frequency | IND1 | N/A | Every week |
|  | LKA1 | N/A | Every week/less than monthly |
|  | MMR1 | N/A | Frequency depends on area |
|  | MMR5 | N/A | Frequency depends on local situation (security, weather and road condition, availability of mobile network etc.) |
|  | NPL1 | N/A | Supervision is not time bound, organised when needed |
|  | THA1 | N/A | Daily |
| Supervision Provider | THA1 | N/A | Community based organisations |
|  |  |  |  |
| Limitations to providing training | AFG | N/A | Coordination and collaboration among managing organizations |
|  | IND2 | N/A | Lack of local language training material, trainers who can provide training in local dialogs, education standard to received technical key points |
|  | KHM1 | N/A | Dropout rate |
|  | LAO | N/A | COVID-19 lockdown restriction |
|  | LKA | N/A | Physical training opportunities lost due to COVID-19. Online trainings were conducted but not effective. e.g. training for malaria microscopy through online sessions is not practical, etc. |
|  | MMR1 | N/A | Accessibility to remote conflict zones |
|  | MMR2 | N/A | COVID-19 restrictions |
|  | MMR4 | N/A | COVID-19 restrictions and Arm Conflicts |
|  | MMR5 | N/A | Formal in-person group trainings were avoided in some implementing townships to avoid gathering in this year 2021 because of coup and COVID. |
| Limitations to providing supervision | AFG | N/A | Difficulty in accessing due to security, route blockage from to snow, floods, cold, mountains, lack of proper roads for vehicles to pass through |
|  | BNG | N/A | COVID-19 lockdown |
|  | KHM2 | N/A | Travel restriction of local health authority due to the COVID-19 pandemic |
|  | LAO1 | N/A | COVID-19 lockdown |
|  | LKA | N/A | Lack of time, cumbersome reporting procedures |
|  | MMR1 | N/A | Access and motivation |
|  | MMR2 | N/A | COVID-19 travel restriction, CHW attritions, lack of time availability from CHW side as well as supervisor side, supervisors are not properly trained for supportive supervision, quality of supervision being conducted |
|  | MMR4 | N/A | Travel restriction due to COVID-19 and Arm Conflict |
|  |  |  |  |
| Programme Evaluation | IND1 | N/A | Impact of fever prevalence rates in the study area amidst declining malaria in the region |
|  | MYS | N/A | Number of reported malaria cases |
|  | THA1 | N/A | Research network |
